# Social Determinants of Health Influence Early Outcomes Following Lumbar Spine Surgery

**DOI:** 10.31486/toj.22.0066

**Published:** 2022

**Authors:** Samuel E. Holbert, Kristina Andersen, Deborah Stone, Karen Pipkin, Justin Turcotte, Chad Patton

**Affiliations:** Department of Orthopedics, Luminis Health Anne Arundel Medical Center, Annapolis, MD

**Keywords:** *Health status disparities*, *orthopedics*, *social determinants of health*, *spine*, *surgical procedures–operative*

## Abstract

**Background:** Disparities among social determinants of health (SDoH) can impact overall well-being and surgical outcomes. The purpose of this study was to identify SDoH for patients who underwent lumbar spine surgery and evaluate their relationship to the postoperative outcomes of length of stay (LOS), discharge disposition, and readmissions.

**Methods:** We conducted a retrospective observational study of patients who underwent lumbar spine surgery from July 2017 to January 2021. We used a self-reported SDoH survey in conjunction with the electronic medical record to gather patient information. Multivariate analysis was used to evaluate the relationships between patient demographics, SDoH, and postoperative outcomes.

**Results:** A total of 951 patients underwent lumbar spine surgery: 484 (50.9%) had decompressive laminectomy alone without fusion, and 467 (49.1%) had decompressive laminectomy with instrumented posterolateral fusion. When controlling for age, American Society of Anesthesiologists physical status classification, and surgery type, the SDoH of being currently married or having a life partner was associated with shorter LOS and decreased likelihood of discharge to a skilled nursing facility. Financial strain was associated with longer LOS, while attending church was associated with a decreased likelihood of 30-day emergency department (ED) return.

**Conclusion:** This study identified various SDoH that may influence postoperative lumbar spine surgery outcomes of LOS, discharge disposition, 30-day ED return, and 30-day readmission. Patients at risk for suboptimal outcomes appear to be those with lower financial resources, less in-home support, and lower social connectivity. Routine screening of SDoH may enable care teams to effectively allocate resources for at-risk patients.

## INTRODUCTION

The surgical treatment of degenerative lumbar conditions can positively impact the quality of life for patients suffering from these pathologies. With an aging population,^1^ the volume of surgical intervention is increasing,^2,3^ and providers and payors must maximize value by controlling costs and optimizing patient outcomes. In addition to biologic and psychologic factors, social determinants of health (SDoH) have been shown to influence a patient's postoperative outcomes, although literature on the topic is scarce.^4^

The World Health Organization (WHO) Commission on Social Determinants of Health report of 2008 brought the concept of SDoH into the public spotlight.^5,6^ According to the WHO, SDoH are broadly defined to encompass the conditions in which people are born and their work and living environments, including degree of economic stability, education, and social and community context.^6^ The US Department of Health and Human Services included SDoH as a major focus of the Healthy People 2030 initiative because SDoH contribute to wide health disparities and inequities.^7^ The Healthy People 2030 initiative identifies 19 distinct SDoH that can be grouped into 5 domains: economic stability, education access and quality, health care access and quality, neighborhood and built environment, and social and community context.^7^ Additionally, a component of racial disparity is typically intertwined with SDoH that influences surgical outcomes such as postoperative complications and length of stay (LOS).^8-10^ Not only do SDoH have a major impact on people's health, well-being, and quality of life,^7^ but SDoH disparities have also been shown to affect clinical outcomes in patients undergoing spine surgery and are associated with an increased risk of poor postoperative outcomes.^11^ Despite this knowledge, little research has focused on the underlying SDoH that may influence outcomes in this patient population.

Our health system, which includes a regional hospital in a suburban setting, routinely collects SDoH data as part of the standard health system intake process. The purpose of this study was to identify SDoH variables in patients who underwent lumbar spine surgery and evaluate their relationship to the postoperative outcomes LOS, discharge disposition, and readmissions.

## METHODS

The institution's clinical research committee deemed this study institutional review board exempt. This retrospective observational study included patients who underwent lumbar spine surgery (either decompressive laminectomy alone or decompressive laminectomy with instrumented posterolateral fusion) from July 2017 to January 2021. Surgeries were performed by 1 of 6 board-certified orthopedic surgeons or neurosurgeons at a single institution. To be included in the study, a patient must have responded to at least 1 question on the self-reported SDoH (SR-SDoH) survey within 2 years of surgery. Patients who had thoracic or cervical surgeries, patients <18 years old at the time of surgery, and protected populations (ie, prisoners and pregnant women) were excluded. Patient consent was deemed unnecessary by the institution's clinical research committee because of the self-report nature of the survey, and no protected health information was used.

### Self-Reported Social Determinants of Health Survey

In July 2017, the SR-SDoH instrument was adopted as a standard tool for gathering SDoH information from patients. The survey includes questions about 10 SDoH. The SR-SDoH is not a validated instrument. The questions were selected from the repository of SDoH questions available through our electronic medical record vendor (Epic Systems Corp). The repository of questions and response scales is derived from previously published, validated instruments.^12-15^ At our institution, the SR-SDoH is available for use in all practice settings but is primarily offered in the health system's primary care offices. The survey is optional, and patients may complete as many of the questions as desired. During the study period, the 951 patients who completed the survey within 2 years of surgery accounted for approximately 40% of lumbar spine surgery patients. We elected to include patients who responded to the SR-SDoH both before and after surgery, given that many SDoH are relatively static over time. The SR-SDoH survey questions and response options are presented in the Appendix.

### Other Independent Variables

In addition to the survey responses, we queried the electronic medical record to gather demographic data including age, race, and American Society of Anesthesiologists (ASA) physical status classification that was used as a proxy for comorbidity burden. We further classified patients by the type of surgery performed, either decompressive laminectomy alone without fusion or decompressive laminectomy with instrumented posterolateral fusion. We extracted the ZIP Codes of residence for all patients and cross-referenced them against external census data to map a median household income (HHI) to each ZIP Code and to identify patients residing in ZIP Codes with a median HHI below the Maryland state median. HHI analysis was not performed for 27 patients residing outside of Maryland.

### Outcome Measures

We examined 4 outcomes commonly used to gauge the quality and efficiency of hospitalization for lumbar spine surgery: LOS (measured in hours), discharge disposition to a skilled nursing facility (SNF), rate of 30-day ED return, and rate of 30-day readmission.

### Statistical Analysis

Descriptive statistics were used to evaluate the demographics, surgeries performed, outcomes, and prevalence of each SDoH measured on the SR-SDoH survey. For descriptive and inferential statistics, the prevalence of each SDoH is presented as a percent of patients responding to that question; nonresponses are not counted for that question. Univariate analysis using the chi-square test or Fisher exact test, when indicated, was performed to compare rates of the various SDoH between White and non-White patients and patients residing in a ZIP Code below or above the median HHI in Maryland. Multivariate linear and logistic regression models were then constructed to evaluate the association between each SDoH and the 4 outcomes after controlling for demographics and procedure type. To avoid interaction effects between the individual SDoH and multiple comparisons, separate models were created to evaluate the relationship between each SDoH and outcome measure after controlling for age, ASA classification, and surgery type. In total, 44 separate models were generated, 1 model for each outcome for each of the 11 SDoH. All statistical analysis was performed in SPSS Statistics, version 27 (IBM Corporation), and statistical significance was assessed at *P*<0.05.

## RESULTS

A total of 951 patients underwent lumbar spine surgery: 484 (50.9%) underwent decompression alone without fusion, and 467 (49.1%) underwent decompression and instrumented posterolateral/interbody fusion. The mean age of patients was 61.2 ± 14.2 years at the time of surgery, 43.4% had an ASA classification ≥3, and 12.7% were of a non-White race. Of the 924 patients residing in Maryland, 161 (17.4%) resided in a ZIP Code with a median HHI above the Maryland state median. The average LOS was 46.7 ± 47.0 hours, and 6% of patients were discharged to an SNF rather than home. Regarding 30-day unplanned revisits, 6.8% returned to the ED and 2.9% were readmitted (Table 1).

**Table 1.** Summary of Patient Demographics, Surgeries, and Outcomes

Variable	All Patients, n=951
Age, years, mean ± SD	61.2 ± 14.2
American Society of Anesthesiologists physical status classification ≥3	413 (43.4)
Non-White race^a^	117 (12.7)
Above the Maryland median household income^b^	161 (17.4)
Surgery performed	
Decompressive laminectomy without fusion	484 (50.9)
Decompressive laminectomy with instrumented posterolateral fusion	467 (49.1)
Outcomes	
Length of stay, hours, mean ± SD	46.7 ± 47.0
Discharge to skilled nursing facility	57 (6.0)
30-day emergency department return	65 (6.8)
30-day readmission	28 (2.9)

^a^Twenty-seven patients chose not to disclose race and are not included.

^b^Twenty-seven out-of-state patients are not included in the household income analysis.

Note: Data are presented as n (%) unless otherwise indicated.

The prevalence of each of the 10 SDoH is presented in Table 2. The most common SDoH reported were the following: currently married or has a life partner (74.6%), above high school education (72.4%), and attends church (54.2%). The least common SDoH reported were the following: has any transportation problem (3.1%), experienced spousal abuse (3.3%), and experienced high levels of stress (7.2%).

**Table 2.** Prevalence of Social Determinants of Health

Social Determinant	Patient Responses, n	Prevalence, n (%)
Exercises 3 or more times per week	280	122 (43.6)
High levels of stress	306	22 (7.2)
Attends church	253	137 (54.2)
Currently married or has a life partner	283	211 (74.6)
Experienced spousal abuse (physical, emotional, or sexual)	273	9 (3.3)
Financial strain (somewhat hard or worse)	294	58 (19.7)
Above high school education	225	163 (72.4)
Has any food worry	292	35 (12.0)
Has any transportation problem	295	9 (3.1)
Drinks alcohol 4 or more days per week	816	129 (15.8)

When comparing SDoH by race, non-White patients were more likely to self-report that they attend church (*P*=0.025) or have any food worry (*P*=0.022) but were less likely than White patients to report drinking alcohol 4 or more days per week (*P*=0.013). When comparing SDoH by HHI, patients residing in ZIP Codes with an HHI below the state median reported higher rates of exercising 3 or more times per week (*P*=0.020) and of drinking alcohol 4 or more days per week (*P*=0.001) (Table 3).

**Table 3.** Social Determinants of Health by Race and Household Income

	Race			Household Income		
Social Determinant	White Race	Non-White Race	*P* Value	Below Median	Above Median	*P* Value
Exercises 3 or more times per week	109/249 (43.8)	13/31 (41.9)	0.846	108/227 (47.6)	14/48 (29.2)	**0.020**
High levels of stress	16/268 (6.0)	5/38 (13.2)	0.158^a^	20/249 (8.0)	2/50 (4.0)	0.551^a^
Attends church	115/223 (51.6)	22/30 (73.3)	**0.025**	119/213 (55.9)	15/37 (40.5)	0.084
Currently married or has a life partner	190/250 (76.0)	21/33 (63.6)	0.125	169/231 (73.2)	35/47 (74.5)	0.853
Experienced spousal abuse (physical, emotional, or sexual)	7/243 (2.9)	2/30 (6.7)	0.259^a^	8/223 (3.6)	1/45 (2.2)	1.000^a^
Financial strain (somewhat hard or worse)	47/256 (18.4)	11/38 (28.9)	0.126	50/239 (20.9)	8/49 (16.3)	0.465
Above high school education	145/200 (72.5)	18/25 (72.0)	0.958	133/182 (73.1)	27/39 (69.2)	0.626
Has any food worry	25/256 (9.8)	9/36 (25.0)	**0.022** ^a^	31/237 (13.1)	4/48 (8.3)	0.361
Has any transportation problem	6/258 (2.3)	3/37 (8.1)	0.090^a^	8/240 (3.3)	1/49 (2.0)	1.000^a^
Drinks alcohol 4 or more days per week	122/719 (17.0)	7/97 (7.2)	**0.013**	117/668 (17.5)	10/149 (6.7)	**0.001**

^a^Fisher exact test. Fisher exact test was performed when >20% of cells had an expected count <5. Chi-square test was performed in analyses without superscript notation.

Notes: Data are presented as number of patients with the social determinant of health/number of eligible respondents (eg, patients responding to the social determinant of health question and with race or household income data available). Statistically significant values are shown in bold.

Multivariate linear and logistic regression models were used to assess the association of each SDoH and the 4 outcome measures after controlling for age, ASA classification, and surgery type. After risk adjustment, being currently married or having a life partner was associated with a shorter LOS (β=–23.4 hours, *P*<0.001) and decreased likelihood of a discharge to an SNF (odds ratio [OR] 0.183, *P*=0.005). Conversely, financial strain was associated with longer LOS (β=14.3 hours, *P*=0.022), while residing in a ZIP Code above the median HHI was associated with increased odds of discharge to an SNF (OR 2.216, *P*=0.018). Regarding unplanned returns, attending church was the only SDoH associated with a decreased likelihood of 30-day ED return (OR 0.250, *P*=0.021) (Table 4).

**Table 4.** Multivariate Linear and Logistic Regression of Outcomes by Household Income Status and Social Determinants of Health After Controlling for Age, American Society of Anesthesiologists Classification, and Surgery Type

Household Income Status/Social Determinant	LOS, Hours, β	Discharge to SNF, Odds Ratio	30-Day ED Return, Odds Ratio	30-Day Readmission, Odds Ratio
Above the Maryland median household income	–3.6 (*P*=0.313)	**2.216** **(*P*=0.018)**	0.576 (*P*=0.180)	0.364 (*P*=0.174)
Exercises 3 or more times per week	–8.1 (*P*=0.105)	0.218 (*P*=0.058)	1.277 (*P*=0.631)	1.118 (*P*=0.871)
High levels of stress	4.3 (*P*=0.653)	0.000 (*P*=0.998)	0.000 (*P*=0.998)	3.004 (*P*=0.217)
Attends church	5.9 (*P*=0.261)	0.776 (*P*=0.675)	**0.250** **(*P*=0.021)**	1.402 (*P*=0.602)
Currently married or has a life partner	–**23.4** **(*P*<0.001)**	**0.183** **(*P*=0.005)**	0.908 (*P*=0.859)	0.701 (*P*=0.573)
Experienced spousal abuse (physical, emotional, or sexual)	–10.2 (*P*=0.452)	0.000 (*P*=0.999)	0.000 (*P*=0.999)	0.000 (*P*=0.999)
Financial strain (somewhat hard or worse)	**14.3** **(*P*=0.022)**	0.000 (*P*=0.997)	2.285 (*P*=0.133)	0.754 (*P*=0.732)
Above high school education	–6.5 (*P*=0.297)	0.834 (*P*=0.783)	0.566 (*P*=0.357)	>100 (*P*=0.997)
Has any food worry	0.7 (*P*=0.927)	0.858 (*P*=0.889)	1.558 (*P*=0.513)	0.000 (*P*=0.998)
Has any transportation problem	21.1 (*P*=0.135)	5.820 (*P*=0.140)	2.338 (*P*=0.450)	3.469 (*P*=0.281)
Drinks alcohol 4 or more days per week	–1.5 (*P*=0.704)	0.498 (*P*=0.138)	0.635 (*P*=0.316)	0.656 (*P*=0.509)

Note: Statistically significant values are shown in bold.

ED, emergency department; LOS, length of stay; SNF, skilled nursing facility.

## DISCUSSION

Our study identified several SDoH that may be correlated with favorable outcomes. When controlling for age, ASA classification, and surgery type, we found that of the SDoH examined, financial resources and strong support systems (ie, currently married or having a life partner) appeared to be correlated with improved postoperative outcomes. Patients with an HHI above the state median were more likely to be discharged to an SNF, while patients with financial strain were more likely to have a longer LOS. Conversely, married patients or those with a life partner were less likely to be discharged to an SNF and had a shorter LOS. Church attendance was the only SDoH associated with a decreased likelihood of a 30-day ED return. Based on these results, patients at risk for suboptimal outcomes appear to be those with lower financial resources, less in-home support, and lower social connectivity (ie, do not attend church). These factors should be considered when preoperatively assessing patient risk profiles; triaging these patients to appropriate support services may improve surgical outcomes.

While a relative paucity of literature evaluating the impact of SDoH on spine surgery outcomes exists, the number of studies evaluating the prognostic value of SDoH has increased. In a 2021 retrospective cohort study, Khalid et al compared 8,280 patients with an SDoH disparity to those without and found that patients undergoing lumbar fusion surgery with at least one SDoH disparity had a 1.7 times increased risk of developing pseudoarthrosis or any other complication.^11^ However, Khalid et al found no difference in revision surgery or 30-day readmission as in our current study. In another study assessing the relationship between SDoH and readmissions, Mohanty et al evaluated 2,830 patients undergoing spine surgeries from 2012 to 2018 in a 3-hospital US metropolitan health network.^16^ In a risk-adjusted analysis, patients with estimated incomes <$31,000 and those residing in neighborhoods with higher diabetes prevalence and limited access to primary care providers were at increased risk for 30-day readmission. Further, each decile increase in the Area Deprivation Index of a patient's census tract was associated with 40% increased odds of readmission.^16^ The Yap et al systematic review of 30,987 adult spine surgery patients from 12 countries provided further evidence of the relationship between SDoH and outcomes.^17^ Seventy percent of analyses identified significant independent relationships between lower education and poorer outcomes, while 67% revealed an independent relationship between lower socioeconomic status and poorer outcomes, leading to the conclusion that these factors are clear independent contributors to poorer outcomes.^17^ A notable strength of the Yap et al study is its focus on functional outcomes, primarily pain and disability as measured by the Oswestry Disability Index, a validated patient-reported outcome measure.^18,19^ However, the clinical and methodological heterogeneity of the studies included in the systematic review precluded the authors’ ability to perform a quantitative meta-analysis.

While these results demonstrate the importance of specific SDoH, others have found that aggregated SDoH risk may hold greater prognostic value than the presence of individual risk factors. Using a national US spine registry, Rethorn et al demonstrated that clusters of SDoH outperformed individual SDoH in predicting clinically meaningful improvements in disability, back pain, leg pain, quality of life, and patient satisfaction at 3 and 12 months following lumbar spine surgery.^4^ The study found that younger, minority, lower socioeconomic status patients were least likely to achieve clinically meaningful improvement in each of these outcome measures. These findings highlight the interconnected nature of SDoH and demographics and provide a methodological framework that may inform future approaches to SDoH research.

Collectively, the results of the studies described largely align with those of our current study. However, a strength of our study is its use of more granular SDoH data, expanding upon the commonly reported importance of socioeconomic status, race, and education. In addition to these factors, our results suggest in-home support and social connectivity may play a role in identifying patients at risk for suboptimal outcomes.

Racial disparity is considered an SDoH. In our study, we did not observe racial disparities; however, previous studies have illustrated differences in outcomes among White and non-White orthopedic patients. Stone et al examined the outcomes of 7,208 patients undergoing either total knee arthroplasty or total hip arthroplasty and found that African American patients had poorer outcomes for LOS and discharge disposition compared to White patients.^20^ Sanford et al analyzed 4,803 spine surgery patients in the American College of Surgeons National Surgical Quality Improvement Program database and demonstrated that African American patients had a longer hospitalization by a full day and higher rates of postoperative complications, including deep vein thrombosis, pulmonary embolism, and surgical site infections, in comparison to White patients.^10^ Clearly, additional research across wide patient populations is needed to better understand the impact of racial disparities following various orthopedic surgeries.

Physicians and care teams need to assess patient health through a wide lens to optimize outcomes following surgery. Preoperative optimization may include both medical and social risk reduction, such as making healthy lifestyle choices before and after surgery and building a strong support system for postoperative care. An investment in nurse navigators could be a potential asset for connecting patients to public programs that can best serve them. Managing the complexities of SDoH, however, will require a team effort including housing authorities, food banks, and even schools, in addition to medical providers.^21^ Community programs that provide lifestyle and financial counseling have a potential benefit for patients and health care institutions alike, as the economic value of reducing SDoH disparities is as much as $1 trillion.^21-23^ On a national level, the Healthy People 2030 initiative addresses SDoH in one of its 5 overarching goals: “Create social, physical, and economic environments that promote attaining the full potential for health and well-being for all.”^7^ This initiative collects data and recommends interventions that can be implemented on a community level to combat disparities within SDoH. The implementation of these strategies must be combined with long-term efforts to grow community strength, change social and cultural norms, and reduce the cost of healthy behaviors to create long-term sustainability.^23^ While Healthy People 2030 addresses SDoH at the systemic level, our findings may help target interventions for the lumbar spine surgery population specifically.

This study was limited by its retrospective design at a single institution and thus may not be representative of the larger population. Furthermore, the inclusion criteria only required patients to have responded to a single question on the SR-SDoH questionnaire, resulting in incomplete data from some patients. Additionally, our inclusion of patients responding to the survey within 2 years of surgery introduced the risk that SDoH status changed during the study period or that surgery influenced patient behaviors. Further, the SR-SDoH instrument used in this study is not a validated measure. To enhance the reproducibility of this study and future SDoH studies, increased utilization of standardized instruments is required. To date, the lack of consensus definitions and standards for capturing SDoH data remains a challenge that has limited the rigor and reproducibility of studies related to the topic.^24,25^

Despite these limitations, these results present a quantitative view of complex SDoH data in a spine patient population. Future efforts will likely include standard definitions, measures, and methods to capture data given the broad scope of SDoH, as well as its subjective definitions. Although we examined 10 distinct SDoH, other variables may have an impact on postoperative outcomes and should be investigated. Specific aspects of SDoH that were not evaluated in this study include health literacy, neighborhood crime and violence, and regional differences in access to health services.^26^ Further, while multiple studies have evaluated the impact of overall comorbidity burden, specific comorbidities, opioid use, and patient-reported function on postoperative outcomes, the interaction between these factors and SDoH has not been explored.^27-32^ Given the interconnected nature of traditional risk factors and SDoH, combining these variables to develop phenotypes of patients at risk for suboptimal outcomes after spine surgery is an opportunity for future research that would be highly applicable in practice. Finally, future studies should evaluate the relationship between SDoH and physical function and mental health status following spine surgery.

## CONCLUSION

This study identifies SDoH that may influence the postoperative lumbar spine surgery outcomes of LOS, discharge disposition, 30-day ED return, and 30-day readmission. Patients at risk for suboptimal outcomes appear to be those with lower financial resources, less in-home support, and lower social connectivity. Routine screening of SDoH may enable care teams to effectively allocate resources for at-risk patients.

## Notes

*This article meets the Accreditation Council for Graduate Medical Education and the American Board of Medical Specialties Maintenance of Certification competencies for Patient Care, *Medical Knowledge*, and Systems-Based Practice.*
